# High-throughput barcoding method for the genetic surveillance of insecticide resistance and species identification in *Anopheles gambiae* complex malaria vectors

**DOI:** 10.1038/s41598-022-17822-8

**Published:** 2022-08-16

**Authors:** Monica Campos, Jody Phelan, Anton Spadar, Emma Collins, Adéritow Gonçalves, Bethanie Pelloquin, Natasha Marcella Vaselli, Anne Meiwald, Emma Clark, Caleb Stica, James Orsborne, Moussa Sylla, Constant Edi, Denka Camara, Abdul Rahim Mohammed, Yaw Asare Afrane, Mojca Kristan, Thomas Walker, Lara Ferrero Gomez, Louisa A. Messenger, Taane G. Clark, Susana Campino

**Affiliations:** 1 Faculty of Infectious and Tropical Diseases, London School of Hygiene and Tropical Medicine, London, UK; 2 Laboratório de Entomologia Médica, Instituto Nacional de Saúde Pública, Praia, 719 Cabo Verde; 3 School of Tropical Medicine and Global Health, University of Nagasaki, Nagasaki, Japan; 4 Swiss Tropical and Public Health Institute, Socinstrasse 57, 4051 Basel, Switzerland; 5 Centre Suisse de Recherches Scientifiques en Cote d’Ivoire, Abidjan, Côte d’Ivoire; 6 Programme National de Lutte Contre le Paludisme, Ministère de la Santé, BP. 595, Conakry, Guinea; 7 Department of Medical Microbiology, University of Ghana Medical School, University of Ghana, Accra, Ghana; 8 Universidade Jean Piaget (UniPiaget), Praia, Cabo Verde; 9 Faculty of Epidemiology and Population Health, London School of Hygiene and Tropical Medicine, London, UK

**Keywords:** Genetics, Molecular biology, Entomology

## Abstract

Surveillance of malaria vector species and the monitoring of insecticide resistance are essential to inform malaria control strategies and support the reduction of infections and disease. Genetic barcoding of mosquitoes is a useful tool to assist the high-throughput surveillance of insecticide resistance, discriminate between sibling species and to detect the presence of *Plasmodium* infections. In this study, we combined multiplex PCR, custom designed dual indexing, and Illumina next generation sequencing for high throughput single nucleotide polymorphism (SNP)-profiling of four species from the *Anopheles (An.) gambiae* complex (*An. gambiae sensu stricto, An. coluzzii, An. arabiensis* and *An. melas*). By amplifying and sequencing only 14 genetic fragments (500 bp each), we were able to simultaneously detect *Plasmodium* infection; insecticide resistance-conferring SNPs in *ace1*, *gste*2, *vgsc* and *rdl* genes; the partial sequences of nuclear ribosomal internal transcribed spacers (ITS1 and ITS2) and intergenic spacers (IGS), Short INterspersed Elements (SINE), as well as mitochondrial genes (*cox1* and *nd4*) for species identification and genetic diversity. Using this amplicon sequencing approach with the four selected *An. gambiae* complex species, we identified a total of 15 non-synonymous mutations in the insecticide target genes, including previously described mutations associated with resistance and two new mutations (F1525L in *vgsc* and D148E in *gste2*). Overall, we present a reliable and cost-effective high-throughput panel for surveillance of *An. gambiae* complex mosquitoes in malaria endemic regions.

## Introduction

Malaria, a mosquito-borne disease caused by *Plasmodium* parasites, is an important public health problem. In the last decade, malaria elimination strategies have contributed to a global fall in incidence rates. However, infection control progress has recently stalled with no further reductions in malaria-attributable mortality, and even reversal in some regions^1^. There were 241 million cases and 627,000 deaths reported in 2020 alone, predominantly in Sub-Saharan Africa^1^. Vector control strategies are based on two primary interventions, namely, insecticide-treated nets (ITN) and indoor residual spraying (IRS). The World Health Organization (WHO) recommends five chemical classes of insecticides (neonicotinoids, carbamates, organochlorines, organophosphates and pyrethroids) for IRS. Whilst for ITNs only pyrethroid insecticides are currently being used alone or in combination with a second active ingredient (the synergist piperonyl butoxide, the pyrrole chlorfenapyr or the juvenile growth hormone inhibition pyriproxyfen). These approaches have contributed significantly to a decline of malaria incidence. However, the effectiveness of vector control is threatened by increasing insecticide resistance in multiple *Anopheles* vectors, which has now been documented in almost all African countries^2,3^.

One of the main mechanisms associated with insecticide resistance is decreased target site sensitivity. Resistance to pyrethroids and DDT have been correlated with target-site mutations in the voltage-gated sodium channel (*vgsc*) gene, also known as knock down resistance (kdr), as well as with metabolic resistance due to point mutations in the glutathione-s-transferase *epsilon* (*gste2*) gene^4,5^. Point mutations and duplications in the acetylcholinesterase 1 (*ace1*) gene have been associated with resistance to both carbamates and organophosphates, whereas mutations in the gaba receptor (*rdl*) have been associated with resistance to organochlorines, particularly dieldrin^6–9^. Other insecticide resistance mechanisms, such as metabolic resistance, mosquito microbiome components and cuticle alterations have also been reported^10–13^, but the molecular mechanisms involved are more difficult to ascertain and monitor at scale.

Current standard methods of identifying phenotypic insecticide resistance involve the use of susceptibility bioassays. However, different approaches are applied across the world, their implementation can be time consuming, the interpretation of results can be subjective, and direct comparison across methods is difficult^14^. Molecular methods for the detection of known mutations associated with insecticide resistance have been developed and can be an effective approach to monitor the emergence and spread of resistance alleles^15^. These assays are mostly based on PCR- RFLP, real-time PCR, or fragment sequencing using Sanger sequencing, which in general only target a few markers^4,16,17^.

Another essential step in designing vector control strategies is the identification of *Anopheles* species, with different major vectors having variable vectorial capacity and different resting or feeding behaviours, and their inference informs tailored interventions for effective control. In Africa, where 94% of all malaria cases and deaths occur, transmission is mainly caused by *An. gambiae sensu stricto (s.s.)*, *An. coluzzii*, *An. arabiensis* and *An. funestus* s.s. The first three species belong to the *An. gambiae* complex, which includes nine morphologically indistinguishable siblings of non-vector and human malaria vector species^18^. As morphological identification alone is not sufficient to separate the sibling species of the same complex, molecular techniques are often required^19^. Molecular assays for species identification and phylogenetic analysis include the internal transcribed spacers (ITS1 and ITS2) and intergenic spacer (IGS) of the ribosomal DNA, the insertion polymorphism of Short INterspersed Elements retrotransposon (SINE200), and mitochondrial genes (*cytochrome c oxidase subunit 1, cox1*; *NADH dehydrogenase 4, nd4*) as these are maternally inherited and lack recombination^20–25^. Linked to species identification is the monitoring of the intensity of malaria transmission by mosquito populations. This surveillance activity facilitates the estimation of the risk of human exposure to the bites of infective anopheline vectors, an understanding of the transmission patterns across regions and seasons, and the vector identification and incrimination.

Given the recent innovations and cost reductions in molecular techniques, screening for insecticide resistance and species identification is likely to be an effective approach to support vector monitoring. Here we designed and validated multiplex PCR assays combined with custom designed dual indexing barcodes and Illumina sequencing for targeted high throughput sequencing of genetic loci related to: (a) insecticide resistance; (b) species identification and mitochondrial barcoding of *An. gambiae* complex mosquitoes; and (c) detection of *Plasmodium* malaria parasites. Amplicon sequencing is a targeted next generation sequencing method that allows for the high-throughput detection of known and new mutations in genomic regions of interest. To demonstrate the utility of our approach we applied it to malaria vectors of four species from the *An. gambiae* complex. Using this method, it is possible to screen many samples across several loci simultaneously, and still discriminate individual samples based on unique barcodes, similar to other methods described for *Plasmodium falciparum*^26^ and diversity investigations within the *Anopheles* mosquito genus^27^. By increasing throughput, efficiency and decreasing costs, this method is a promising tool to support malaria vector control surveillance.

## Results

### Anopheles gambiae s.l. molecular species identification and geographic separation

A total of 110 female *An. gambiae s.l.* mosquitoes were analysed, from which 93 (84.5%) DNA samples passed quality control filters. Species identification was performed using results from the amplicon assays for IGS and SINE200 and compared to morphological speciation by classical taxonomy and standard molecular methods. An in-house script (see “Methods”) for species identification was developed based on data from species-specific nucleotide sequences for the IGS and an insertion in the SINE200 amplicon previously described to be fixed in all M (*An. coluzzii)* and absent in all S specimens (*An. gambiae s.s.)*^20^. Using a polymorphic region in the IGS, the script was able to discriminate between *An. arabiensis*, *An. melas* and *An. gambiae* (*coluzzii* or *s.s.*), matching morphological data (Table 1; Supplementary Table 1). Further, SNPs in the *gste2* amplicon allowed the separation of *An. arabiensis* from the remaining species (Supplementary Tables 1, 2). Data from *An. arabiensis* samples from other geographic regions are needed to confirm this finding.

**Table 1:** Species identification using intergenic spacers (IGS) and Short INterspersed Elements (SINE200) for the 93 samples.

Country	Collection year	Species calling	N
Cabo Verde	2017	*An. arabiensis*	24
Ghana	2015	*An. melas*	2
Guinea	2017/18	*An. gambiae s.s*	19
Guinea	2017/18	*An. coluzzii*	5
Ivory Coast	2019	*An. gambiae s.s*	8
Ivory Coast	2019	*An. coluzzii*	15
Ivory Coast	2019	Hybrid	7
Ivory Coast	2019	Hybrid	1
Kenya	Insectary samples	*An. gambiae s.s*	12

The country, date of collection and *An. gambiae complex* species are shown. The last column (N) contains the total number of samples per species detected in each country.

Hybrid = *An. coluzzii*/*An. gambiae s.s*.

We PCR-amplified the SINE200 segment of 29 randomly selected samples and analysed by electrophoresis gel to check for a band of size 249 bp for *An. gambiae* s.s. and 479 bp for *An. coluzzii* molecular forms. If both bands were present, samples were called as hybrids. These data were compared with the amplicon sequencing results, and a match of 100% (29/29) was observed. The in-house script detected 21 *An. gambiae s.s.,* 3 *An. coluzzii* and 5 hybrids (Supplementary Table 1). An additional 64 samples underwent amplicon sequencing. From mosquitoes captured in Guinea (n = 24), 79% were identified as *An. gambiae s.s.,* and 21% were *An. coluzzii*. Samples from Ivory Coast (n = 31) were determined to be *An. gambiae s.s.* (26%), *An. coluzzii* (48%), hybrids (23%), and for the remainder (3%) it was not possible to distinguish between *An. gambiae coluzzii or s.s.* species due to low coverage in the SINE200 amplicon. Finally, Kisumu strain samples (originally from Kenya) from the LSHTM insectary were confirmed to be *An. gambiae s.s*.

**Figure 1:**
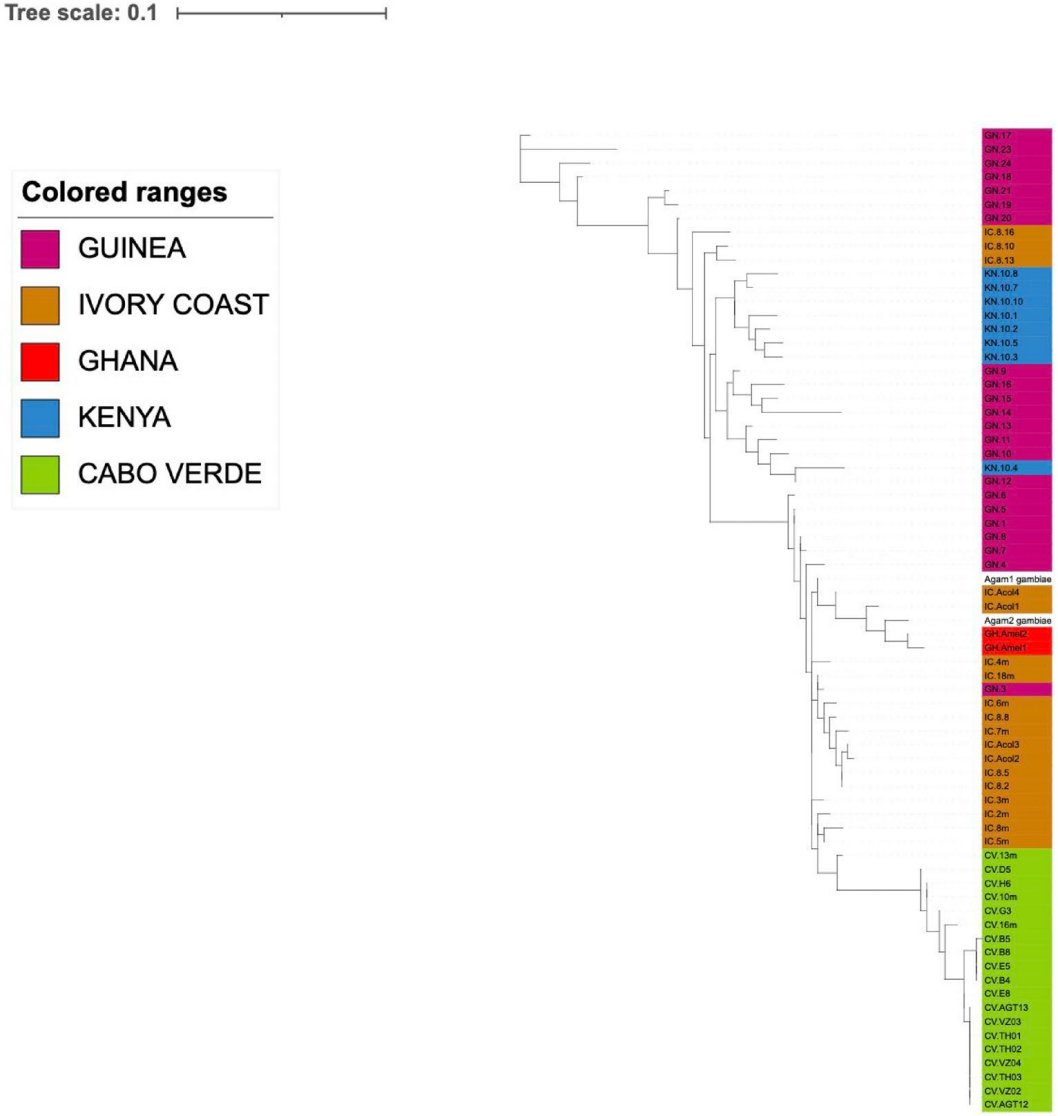
Phylogenetic tree of the nuclear region *ITS1*. The tree was constructed using a maximum likelihood model (GTR + G) with a bootstrap node support of 100 replicates. Sample names are shown around the edge of the tree, colored by country of origin: brown (Ivory Coast), green (Cabo Verde), red (Ghana), Pink (Guinea) and blue (Kenya).

The amplified complete sequences of the ribosomal DNA internal transcribed spacers ITS1 and ITS2 were also analysed to investigate genetic variation across species and geographic regions. For ITS1, sequence length variation of < 30 bp was detected among samples from the same country (Guinea), whereas for ITS2, the maximum intra-specific variation in sequence length was 5 bp (Ivory Coast and Guinea). A total of 24 SNPs were detected in ITS1 and 6 SNPs in ITS2 (Supplementary Table 3). Phylogenetic analyses were performed separately for each genomic target. Both trees contain the *An. arabiensis* sequences from Cabo Verde separated in a clear clade from the remaining samples. When using the ITS1 genomic region, there was greater resolution to a country level (Fig. 1, Supplementary Fig. 1). One clade consisted of samples from Ivory Coast (15/18), Guinea (6/22) and Ghana (n = 2; *An. melas*). A second clade includes all the samples from the Kisumu colony, three samples from Ivory Coast (3/18) and most samples from Guinea (16/22).

The mitochondrial genes *cox1* and *nd4* were also analysed to investigate the phylogenetic relationships of these species and the geographic resolution. Sequencing data analyses revealed a total of 66 SNPs, including six non-synonymous mutations (*cox1* S333R, F346C; *nd4* G327R, R288S, V375F and I218M) (Supplementary Table 3). Phylogenetic analyses using both loci revealed a clade that separated most of *An. arabiensis* (Supplementary Figs. 2, 3) with additional clustering similar to the ITS2-based analyses, with ITS1 showing the best geographic resolution across the four loci investigated (Fig. 1).

### Detection of SNPs in genes associated with insecticide resistance

**Figure 2:**
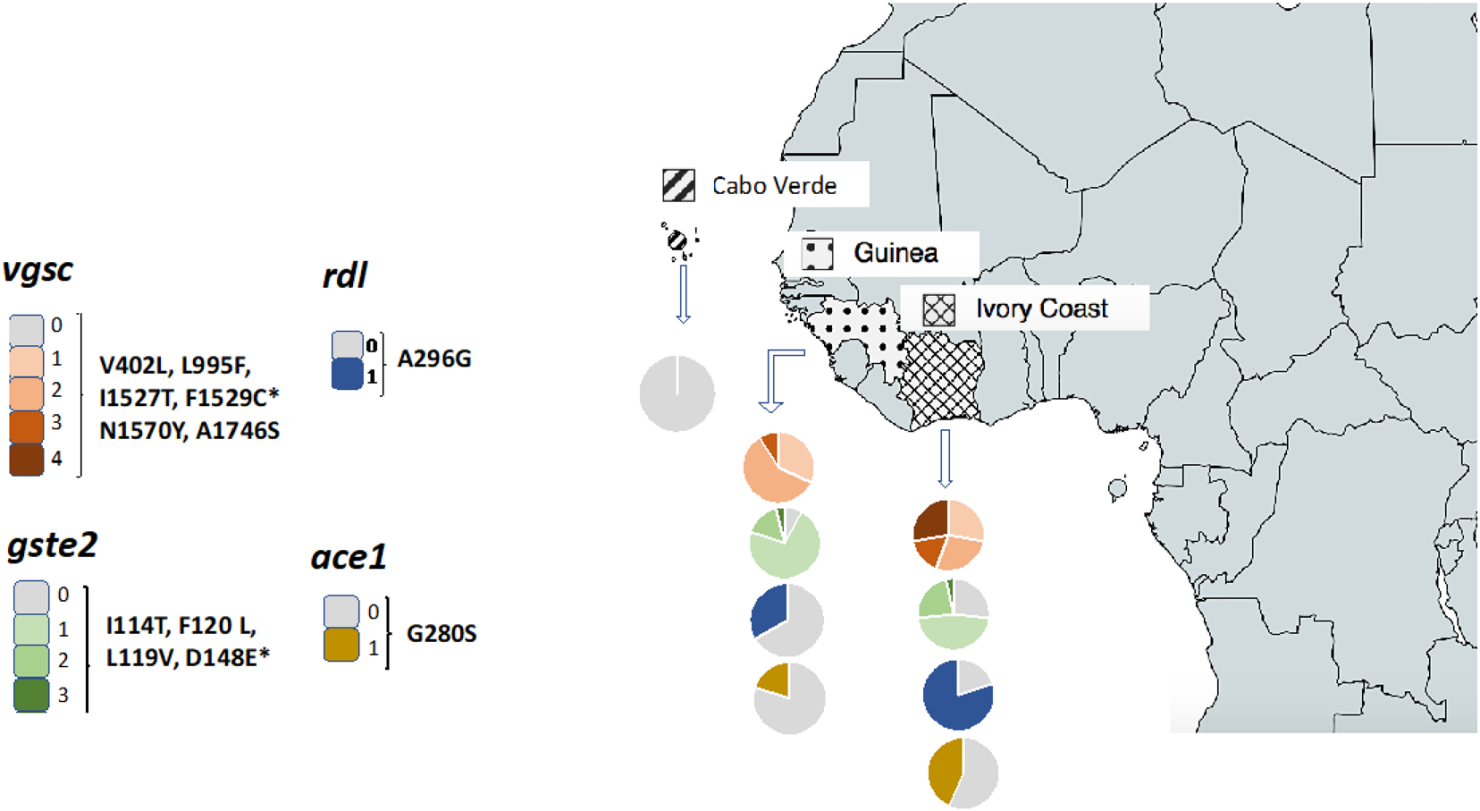
Number of insecticide resistance mutations detected in *Anopheles gambiae* s.l. from Guinea, Ivory Coast and Cabo Verde. The pie chart represents the percentage of samples from each country harboring zero, one or more mutations in each gene: *vgsc* (1–4) in red gradient, *gste2* (1–4) in green gradient, *rdl* (1) in blue, *ace1* (1) in amber. Mutations were called in accordance with the amino acid positions in *An. gambiae*. The correspondent amino acid positions in *Musca domestica* are shown in brackets: V402L (V410), L995F (L1014), I1527T (I1532), F1529C (F1534), N1570Y (N1575), A1746S (A1751), A296G (A282), I114T (L114), F120L (Y120), L119V (L119), D148E (H148), G280S (known as G119). *Indicates missense mutations newly described for the *Anopheles* genus.

A total of 133 SNPs were detected across the seven amplicons designed to target for loci (*vgsc, rdl, gste2, ace1*) associated with insecticide resistance. These included 66 SNPs in exons (15 non- synonymous), 56 SNPs in introns, 10 SNPs in untranslated regions, and one SNP in a splicing region (Table 2, Supplementary Table 4). Most non-synonymous SNPs have been previously linked with insecticide resistance. For the *vgsc* gene, four amplicons were designed (*vgsc*-DIS6, -DIIS6, -DIIIS6, -DIVS5). In the *vgsc*-DIS6 amplicon, a total of 16 SNPs were identified, and included the V402L substitution detected in *An. gambiae s.s.* and *An. coluzzii* samples from Guinea (3/21) and Ivory Coast (7/14) (Supplementary Table 5). In the *vgsc*-DIIS6 amplicon, a total of 5 SNPS were found, including the L995F substitution (995F resistance—R; 995L sensitive—S), which was detected in mosquitoes from Guinea (67% R/R; 33% R/S) and Ivory Coast (57% R/R; 36% R/S). In the *vgsc*-DIIIS6 amplicon, we detected a total of 22 SNPS with four amino acid substitutions, including three known insecticide resistance mutations (I1527T, F1529C and N1570Y). The 1527 T mutation was detected in samples from Guinea (4%) and Ivory Coast (36%). Whilst the F1529C mutation was only found in samples from Ivory Coast (44%), and N1570Y was detected in Guinea (36%) and Ivory Coast (4%). A new mutation F1525L was found in Guinea (68%) and Ivory Coast (28%). In the *vgsc*-DIVS5 amplicon. A total of three new SNPs were detected, including the A1746S mutation, which was present in samples from Guinea (24%) and Ivory Coast (7%) (Fig. 2; Supplementary Table 5).

**Table 2:** Distribution of SNPs per amplicon.

Locus	Syn	Non-syn	Intron	Splice site	3′-UTR	Total
vgsc-DIS6	0	1	15	0	–	16
vgsc-DIIS6	2	1	2	0	–	5
vgsc-DIIIS6	6	4	13	1	–	24
vgsc-DIVS5	3	1	0	0	–	4
*rdl*	0	1	18	0	–	19
*gste2*	16	6	9	1	8	40
*ace1*	24	1	–	–	–	25
Total	51	15	56	1	10	133

Syn: synonymous, Non-syn: non-synonymous, 3′-UTR: 3 prime untranslated region. The amplicons are represented as follow: voltage gate sodium channel (*vgsc*) domains (D) and subunits (S); *rdl; gste2* and *ace1* genes.

For the *rdl* gene, the amplicon designed allowed for the detection of 19 SNPs, including one non-synonymous substitution A296G, which was found in *An. gambiae* samples from Guinea (21% R/S) and Ivory Coast (55% R/S, 25% R/R) (Fig. 2; Supplementary Table 5). Similarly, for the *gste2* gene, the single amplicon allowed the detection of 42 SNPS, with six missense mutations, including three associated with insecticide resistance (I114T, F120L, L119V) (Fig. 2; Supplementary Table 5). The I114T mutation was found in *An. gambiae* samples from Guinea (29%), Ivory Coast (20%) and Ghana (n = 1; *An. melas*). The substitution L119V was detected in Guinea (50%) and Ivory Coast (27%). The F120L mutation was found in samples from Guinea (21%), Ivory Coast (63%) and Ghana (50%). Whilst V131L was found exclusively in *An. arabiensis* samples from Cabo Verde (54%). The T154S substitution was detected in the Kisumu wild type samples, as well as in Guinea (50% R/S, 21% R/R) and Ivory Coast (47% R/S, 17% R/R). In addition, a new mutation D148E was detected in *An. coluzzii* samples (20%) from Guinea. Lastly, for the single amplicon of the *ace1* gene, 25 SNPs were found, including one non-synonymous G280S mutation associated with insecticide-resistance, which was present in samples from Guinea (17% R/S) and Ivory Coast (37% R/S, 3% R/R) (Fig. 2; Supplementary Table 5).

For the *vgsc* gene, all samples from Ghana and Ivory Coast had at least one SNP associated with resistance, 58% of samples from Guinea presented two mutations, and 28% of the samples from Ivory Coast harbored four SNPs (Fig. 2). For the *gste2* gene, a total of three missense mutations linked to resistance were identified, and 72% of samples from Guinea had one SNP, while 4% had a combination of three SNPs. In Ivory Coast, 47% and 3% of samples presented with one and three non-synonymous mutations, respectively.

### Detection of *Plasmodium* infection

*Plasmodium 18S rRNA* was targeted in 16 randomly selected samples from Guinea (n = 8) and Ivory Coast (n = 8). Using amplicon sequencing, we detected (8/16) mosquitoes positive for *P. falciparum.* Most samples were also confirmed to be positive using standard qPCR (overall agreement of 93%; 13/14, 2 samples failed qPCR) (Table 3). The higher the coverage for the targeted region, the lower the cycle threshold obtained in the qPCR assay. For samples with total amplicon coverage below 75-fold, the cycle threshold values after qPCR assays were between 28 and 34 (Table 3).

**Table 3:** Comparison between amplicon sequencing and molecular standard qPCR method for the detection of *Plasmodium* infections in wild-caught *Anopheles gambiae* s.l. samples from Guinea and Ivory Coast.

Sample	Amplicon sequencing (depth)	qPCR (Ct)	qPCR RESULT
S1	0	neg	neg
S2	0	neg	neg
S3	0	neg	neg
S4	0	neg	neg
S5	0	neg	neg
S6	6	neg	neg
S7	45	neg	pos
S8	16	nd	nd
S9	75	34	pos
S10	136	16	pos
S11	176	23	pos
S12	51	nd	nd
S13	15	31	pos
S14	18	33	pos
S15	49	28	pos
S16	66	33	pos

The total depth in amplicon sequencing is shown. Cycle threshold (Ct) values for quantitative PCR assays (qPCR). Results are negative (neg), positive (pos) or not determined (nd).

### Comparison of amplicon sequencing with standard methods and pooling

To assess amplicon sequencing data accuracy when compared with standard methods, the following assays were performed in parallel using target-site mutations for the detection of L995F, N1570Y, and G280S. In the amplicon sequencing, the genotypes were predicted using the ratio of the alternative allele and total allele depths (Supplementary Table 6). For N1570Y, all samples were the homozygous reference allele genotype using both methods (24/24; 100%). For G280S and L995F, four samples were heterozygous genotype in one assay and homozygous non-reference in another (4/29; 86.2%), both assays still detected at least one allele carrying the mutation in these samples. For G280S, one sample was classified as susceptible (S/S) genotype by the standard method but was called heterozygous (R/S) using amplicon sequencing.

The multiplex assay involves the use of unique barcodes per sample, permitting the pooling of many individual samples and their later discrimination in the analysis. Nevertheless, we tested the pooling of samples before amplification, as pooling of samples collected in the same location and time assists with estimating the prevalence of insecticide resistance, reduces DNA extraction costs and labour time (DNA extraction of pools of mosquitoes vs. individual mosquitoes), and increases sample size in a sequencing platform run. Pooling has been previously tested using TaqMan assays and found to be precise in detecting SNPs^28^. The efficacy of sample pooling in relation to SNP detection was verified by pooling DNA samples prior to PCR assays. We compared the results of individual samples to the pooling of five samples (Pool5) prior to amplification (Table 4). Each pool was amplified with a unique barcode to permit further pooling at sequencing step. The resistant alleles detected in each individual sample were also detected after pooling. For instance, for the *gste2* positions 28598062 and 28598136, 1/5 samples were heterozygous (R/S), while 4/5 samples were a homozygous reference allele (S/S) genotype. In each case, one alternative allele (R) in a total of 10 is present in the pooled sample. The genotype prediction for Pool5 for those positions was heterozygous (R/S), confirming the presence of the resistant allele.

**Table 4:** Genotypes of pooled samples versus individual samples.

Chr	Position	Nt change	Gene	Amino acid change	RS	RR	SS	Genotype pool5
Mito	2460	T > G	*cox1*	F346C	2		3	RS
3R	28597956	G > C	*gste2*	T154S		5	0	RR
3R	28598012	C > T	*gste2*	syn		5	0	RR
3R	28598062	G > C	*gste2*	L119V	1		4	RS
3R	28597707	T > A	*gste2*	3-UTR		5		RR
3R	28597715	A > C	*gste2*	3-UTR	5			RS
3R	28597719	T > C	*gste2*	3-UTR		5		RR
3R	28597723	C > G	*gste2*	3-UTR		5		RR
3R	28597729	G > C	*gste2*	3-UTR		5		RR
3R	28598123	A > C	*gste2*	intron	1	4		RS
3R	28598136	G > A	*gste2*	intron	1		4	RS

Individual mosquitoes were processed and submitted for amplicon sequencing. In parallel, DNA aliquots of each sample were pooled together (pool5) and sequenced. SNPs were called for each individual and/or pooled sample and the genotypes detected were shown as RS (heterozygous), RR (homozygous non-reference), and SS (homozygous reference). Chr (chromosome), Nt (nucleotide).

## Discussion

Our work describes a valuable genomic tool for large-scale malaria vector control surveillance strategies that can be applied and extended to simultaneously identify species within the *An. gambiae* complex, detect *Plasmodium* infection*,* and find genetic variants in loci associated with insecticide resistance. Using previously described genetic markers (SINE200, IGS), the amplicon assay differentiated between four *An. gambiae* complex species. By incorporating rDNA nuclear (*ITS1*, *ITS2*) and mitochondria (*cox1, nd4*) genes, previously found to be useful for reconstructing phylogenetic relationships within several vector complexes and cryptic species, our analysis revealed that majority of *An. arabiensis* from Cabo Verde cluster together. The ITS1 locus data provided better species and geographic clustering than the other three loci, consistent with previous work^29^. Further, amplicons covering four loci (*vgsc, rdl, gste2, ace1*) associated with insecticide resistance were evaluated. Most mutations identified in coding regions (51/66; 77%) led to synonymous amino acid changes, with some studies suggesting that they can affect RNA stability, protein folding and cell fitness^29^.

Most of the 15 non-synonymous SNPs identified in our dataset have been previously associated with insecticide resistance (*vgsc* 6/7*, rdl* 1/1*, gste2* 5/6*, ace1* 1/1)^21,30^. For *vgsc*, a new mutation F1525L was found in Guinea and Ivory Coast. The known 995F frequency approaches fixation in Guinea and Ivory Coast (100% and 93%, respectively), and high frequencies of this mutation have been found in west and central Africa and have been linked with secondary selection of other mutations that may enhance or compensate for the L995F phenotype^31^. For example, the N1570Y substitution has been shown to substantially increase pyrethroid resistance when present in combination with L995F^32^, and in our data, the N1570Y frequency was low (< 3.8%) except in Guinea (36%), and always present in the same haplotype as the 995F substitution. We also detected A1746S at low frequencies in Guinea, as previously described^31^, and in Ivory Coast, not previously reported. For *gste2*, a new mutation D148E was found in *An. coluzzii* samples from Guinea. In addition, we detected the mutation L119V, previously reported in *An. funestus* as L119F and associated with high levels of metabolic resistance to DDT^33^*.* Mosquitoes from both Ivory Coast and Guinea also presented substitutions I114T and F120L, with the latter at a high frequency in Ivory Coast and located in close proximity with the putative DDT binding site^34^.

For *rdl*, the 296G mutation was detected in Guinea and Ivory Coast and is the target site for cyclodiene insecticides (e.g., dieldrin), which have been discontinued from use in malaria control programmes. Despite the ban on dieldrin decades ago, molecular analysis studies have shown that despite the A296G substitution declining in some mosquito populations, it has persisted at high frequencies in others^35–39^. Here we show higher allele frequency of A296G mutation in mosquitoes from Ivory Coast when compared with those from Guinea, corroborating a previous report^40^. The 296S substitution has also been associated with current insecticides (e.g. fipronil) in *An. arabiensis, An. funestus,* and *An. sinensis*^7,38,39,41^. In the *ace1* gene, the G280S substitution (G119S in *Musca domestica*), associated with resistance to carbamates and organophosphates, was identified in our Guinea and Ivory Coast samples, and has been reported in the border country Mali, where it is associated with bendiocarb resistance^42,43^. Overall, *An. gambiae s.l.* mosquitoes from Guinea and Ivory Coast were shown to carry resistant alleles to insecticides of all approved classes. Extreme and multiple resistance mosquito populations have been reported in this geographical region, and therefore represent a major threat to malaria control in West Africa^44^. The Cabo Verde samples had no mutations associated with insecticide resistance. Recently, the *kdr* L1014S and L1014F substitutions were detected at low frequencies (10% and 19%, respectively) in *An. arabiensis* collected in Cabo Verde^45^, suggesting it is possible that these mutations are increasing in frequency over time in this region.

Our data revealed that the amplicon method can detect *Plasmodium* infections in a single wild-caught mosquito, as previously reported in human blood samples^26^ and laboratory-infected mosquitoes^27^. More generally, one drawback in amplicon sequencing assays is the low level of primer tagging among samples pooled together in the same run^46^. To overcome possible false positive SNP calling, we set a high threshold for assuming a real SNP while still being able to predict with high confidence the presence of at least one resistant allele in each single mosquito. Using this approach, our findings are consistent with previous data on wild-caught mosquitoes from Ivory Coast^47^ and Guinea^42,48^. Our data also corroborates previous findings in *An. arabiensis* from Cabo Verde, describing the high frequency of V131L mutations in the *gste2* gene^49^. Furthermore, our panel provided information of a broad range of missense mutations that have not been investigated in those previous studies using the same batch of samples.

Previous work on amplicon sequencing has focused on species identification towards *Anopheles* genus and *Plasmodium* infections using a different panel of 62 loci^27^. Here we describe a high-throughput tool for the surveillance of insecticide resistance, species identification and malaria parasite infection detection in malaria vectors from specifically the *An. gambiae* complex using a panel of only 14 genetic fragments. The approach allows the tracking of known mutations across populations and the identification of new genetic variants. Combined with phenotyping, this assay can help identify functional SNPs that are predictive of resistance to a particular intervention/insecticide. The assay is easy to implement and can be applied to many samples at low cost, achieved through PCR multiplexing and dual barcoding. As the PCR stage already includes Illumina-compatible flowcell adaptor sequences, there is no need to prepare libraries for Illumina sequencing, leading to multi-locus amplicon pools that can be sequenced by commercial providers at relatively low cost (< US$ 0.5 per amplicon). Further, the amplicons can also be sequenced in portable sequencers (e.g., Oxford Nanopore Technology MinIon), allowing for vector surveillance at field sites. Large scale monitoring can provide key information to support decision making in malaria vector control programs, which are crucial in reducing disease burden. For example, the discovery of new mutations linked to resistance alongside the screening of well-known markers can lead to changes in insecticide use. New loci linked to resistance, speciation and geographical location can be included as amplicons, thereby further facilitating the tracking of mosquitoes and malaria parasites across populations, to inform vector and disease control.

## Material and methods

### Mosquito sampling

A total of 110 mosquito samples were used in our study, including wild caught and laboratory colonies of the *An. gambiae* complex. Specimens were captured as part of ongoing studies in Guinea (n = 24; years 2017–2018)^42,48^, Ivory Coast (n = 46; 2019)^47^*,* Cabo Verde (n = 24; 2017) and Ghana (n = 2; 2015)^50^. These mosquito collections were chosen to represent genetically diverse members of the *An. gambiae* complex with differing rates of *Plasmodium* infection, distinct insecticide resistance intensities and underlying resistance mechanisms to validate these novel assays. Adult mosquitoes from the *Anopheles* genus were identified by a morphological key^51^. In Ivory Coast, study activities were conducted in the village of Aboudé, rural Agboville, Agnéby-Tiassa region and adult mosquitoes were collected using human landing catches (HLCs)^51^. In Guinea, mosquito collections were undertaken in six villages in the Maferinyah sub-prefecture, Forecariah Prefecture, by manual aspiration from house walls and HLCs^51^. Samples from the city of Praia in Santiago Island in Cabo Verde were collected as larvae in natural and artificial breeding sites. In Ghana, mosquitoes were collected using CDC resting traps from the village of Dogo, in the Greater Accra region of Ghana^50^. *An. gambiae s.s.* Kisumu strains (originally from Kenya) (n = 14) were obtained from the insectary colony at the LSHTM^51^.

### DNA extraction

Individual mosquitoes were disrupted using a Tissue Ruptor II (Qiagen, Hilden, Germany) at speed 3 for sixty seconds. DNA of each single mosquito was extracted using Qiagen DNeasy Blood and Tissue Kits following the manufacturer’s protocol. DNA samples were quantified using the Qubit 2.0 fluorimeter HS DNA kit (ThermoFisher, Waltham, MA, USA) and stored at − 20 °C.

### Primer design and PCR reactions

Sequences were downloaded from VectorBase (https://vectorbase.org/vectorbase/app) and aligned and regions of high homology among species were chosen. *An. gambiae* PEST, *An. coluzzii* Ngousso, *An. melas* CM1001059_A, and *An. arabiensis* reference genome sequences were used in the mapping process. Our high-throughput methodology was designed to amplify a total of 14 genes/genomic regions in the *An. gambiae* complex genome. Seven genomic regions were targeted for species identification and/or phylogenetic analyses: 28S ribosomal RNA and rRNA intergenic spacer region (IGS)^25^, SINE200 (short interspersed elements)^20^, nuclear ribosomal internal transcribed spacers 1 and 2 (*ITS1, ITS2*), cytochrome c oxidase I (c*ox1*), mitochondrially encoded NADH dehydrogenases 4 (*nd4*) (Supplementary Table 7). A total of 17 SNPs associated with insecticide resistance were selected, based on previous reports on the *Culicidae* family (Supplementary table 8). Primers were designed to target 7 genomic regions containing those 17 SNPs: 4 different domains of the voltage-gated sodium channel (*vgsc* DI-IV), partial sequences of the genes acetylcholinesterase 1 (*ace1*), glutathione S- transferase 2 (*gste2*) and resistance to dieldrin gamma-amino butyric acid receptor (*rdl*) (Supplementary Table 9, Fig. 4). The 18S ribosomal RNA of *Plasmodium spp* was also targeted to detect *Plasmodium*-infected mosquitoes (Supplementary Table 9).

For the 1st PCR step, primer design was based on a previous publication with a few modifications^26^, including a 5′tag inline barcode (6 bp long) that was added to each forward and reverse gene target primers. Each primer consisted of 3 regions from 5′: universal Illumina partial adaptors (PE adaptor), inline barcodes with unique combinations per sample and locus specific primer targeting the gene of interest (Supplementary Fig. 5, Table 10). Target DNA sequences (genes/genomic regions) in *Anopheles* species *(An. gambiae s.s* and *An. coluzzii, An. arabiensis and An. melas*) were downloaded from the VectorBase website and aligned using ALIVIEW software^52^. Similarly, the 18S ribosomal RNA sequences of *P. vivax, P. falciparum, P. ovale and P. malariae* were downloaded from the PlasmoDB database (https://plasmodb.org/plasmo/app), aligned and the region of interest was selected. Primer design was performed using the Primer3 program^53^ and the selection criteria for the region of interest was based on three aspects: sequence similarity among all species, the position of SNPs of interest, and amplicon size of approximately 500 bp. Primers were tested using control samples for *P. falciparum*, *P vivax*, *P. malaria* and *P. ovale* from the LSHTM Malaria reference laboratory (Supplementary Fig. 6). The universal adaptors contained non-annealing overhangs on both forward and reverse primers, which were incorporated into both ends of the PCR product during the 1st PCR step, and act as annealing sites for the Illumina indexing primers during the 2nd PCR step. Primers were designed to have similar annealing temperatures in order to be eligible for multiplexing. After designing the primers, we checked for the best combinations using “Multiple Primer Analyzer” software (Thermo Fisher Scientific) to avoid primer dimerization. The 1st PCR reaction (final volume of 25 μl) was performed multiplexing gene-specific primers (0.75 μl/10 μM) as follows: *ITS1, ace1, vgsc*-DIIIS6, *cox1* (multiplex-1); *ITS2*, *nd4*, *rdl*, *vgsc*-DIIS6, (multiplex-2); *vgsc*-DIVS5, *IGS, gste2* (multiplex-3); *vgsc*-DIS6, SINE200 (multiplex-4) and *Plasmodium* ssp (single-plex). Assays were performed using Q5 high fidelity DNA polymerase under the following thermocycler conditions: heat activation at 98 °C for 30 s, 35 cycles of denaturation at 98 °C for 10 s, annealing at 57 °C for 60 s, and elongation at 72 °C for 1 min, and one final elongation at 72 °C for 2 min.

### Amplicon purification and next generation sequencing

After gene-specific multiplex PCR reactions, amplicons were visualized in a 1% agarose gel to check for band size and intensity, and DNA quantification was performed using Qubit dsDNA HS Kit (Thermo Fisher). Each PCR multiplex from different mosquito samples were pooled together, resulting in 4 final tubes (pool_multiplex 1, pool_multiplex 2, pool_multiplex3, pool_multiplex4). The pools were bead purified with AMPure XP beads (Beckman Coulter, California, United States) according to manufacturer’s protocol, using 200 µl PCR product (per pool), and 0.8 × PCR-pool volume of beads (= 120 ul). Concentration of purified multiplexes was measured on a Qubit prior to pooling all the 4 multiplexes in a single tube at a final concentration of 20 ng/µl (25 µl final volume). The final pool containing all PCR products for all mosquito samples was then used as template in the indexing PCR (second PCR step) performed by GENEWIZ and sequenced using the Illumina MiSeq platform (Illumina, San Diego, CA, USA). Sequencing was performed using a paired-end (250 bp) configuration. A minimum of 50,000 reads were obtained per pool (250 reads per amplicon in a pool of 200 amplicons) using the Genewiz service (< US$ 0.5 per amplicon). Sequence reads were trimmed to remove possible adapters and nucleotides with poor quality at 3′ end.

### *Plasmodium falciparum* detection

Samples were tested for the presence of *P. falciparum* using a qPCR assay targeting the cox-1 gene^54^. Reactions were prepared with 1 µl of 10 mM forward (5′TTACATCAGGAATGTTATTGC-3′) and 10 mM reverse (5′-ATATTGGATCTC CTGCAAAT-3′) primers, 1 µl of water, 5 µl of SYBR® Green master mix (Applied Biosystems®, US), and 2 µl of gDNA. The plates were run in the Roche Lightcycler® 96 Real-Time PCR system for 15 min at 95 °C, 35 cycles of 95 °C for 15 s, and 58 °C for 30 s. Fluorescence results were analyzed using the Roche Lightcycler® 96 software.

### Detection of insecticide resistant mutations

To validate sequence results, conventional reactions were used to check for specific insecticide resistant mutations. PCR primers targeting L1014F substitution was carried out following MR4 guidelines (https://www.beiresources.org/Publications/MethodsinAnophelesResearch.aspx) and qPCR was reformed to target the N1570Y mutation^32^. PCR followed by restriction enzyme digestion was used to detect the presence of the *ace1* resistant allele as previously described^16^.

### Bioinformatics analysis

Raw sequences were de-multiplexed using an in-house python script. From the sequenced pool, individual sample data was de-multiplexed based on the in-line barcodes in each forward and reverse primer using an inhouse pipeline (https://github.com/LSHTMPathogenSeqLab/amplicon-seq) to remove any mis-tagging across barcodes. Sequences were trimmed and aligned to the reference genome or the gene sequence fasta files. Sequence data was checked for quality using FastQC (v 0.11.5). Paired end reads were mapped against the reference sequences for the BWA-MEM algorithm (v0.7.17, default parameters^55^). SNPs and small indels were called using freebayes (v1.3.5, -haplotype-length -1) and GATK HaplotypeCaller (v4.1.4.1, default parameters) software. The union of variant sets from both callers was used to produce a list of variants for each sample using a custom python script. High quality SNPs were identified using filters that included a minimum phred quality of 30 per called base, minimum depth of 30 reads, and minimum allele depth of 10. Finally, only SNPs that were present in more than one sample, and present across two independent pools were retained. For each individual sample, the percentage of alternative allele to total depth coverage was used to classify genotypes: (i) homozygous susceptible (S/S; 0% to 25%); (ii) heterozygous (R/S) (25% to 75%); (iii) homozygous non-reference (R/R; 75% to 100%). Visualization of sequence assemblies was carried out using Tablet software. Species identification was performed in samples based on the cut-off established by the comparison assay: *An. coluzzii* SINE200 reads > 10% were *An. coluzzii*; *An*. *coluzzii* reads < 1% sample was *An. gambiae s.s* form; if values lay between both reads, samples were called as hybrid.

## Supplementary Information

Supplementary Information 1.

Supplementary Information 2.

## Data Availability

All raw sequence data is listed in the European Nucleotide Archive (ERR9693161 to ERR9693176).

## References

[1] World Health Orgnization (WHO). *World malaria report 2021*. https://www.who.int/teams/global-malaria-programme/reports/world-malaria-report-2021 (2021).

[2] Hancock PA (2018). Associated patterns of insecticide resistance in field populations of malaria vectors across Africa. Proc. Natl. Acad. Sci. U.S.A..

[3] Moyes CL (2020). Evaluating insecticide resistance across African districts to aid malaria control decisions. Proc. Natl. Acad. Sci. U.S.A..

[4] Martinez-Torres D (1998). Molecular characterization of pyrethroid knockdown resistance (kdr) in the major malaria vector *Anopheles gambiae* s.s. Insect Mol. Biol..

[5] Riveron JM (2014). A single mutation in the GSTe2 gene allows tracking of metabolically based insecticide resistance in a major malaria vector. Genome Biol..

[6] Silva APB, Santos JMM, Martins AJ (2014). Mutations in the voltage-gated sodium channel gene of anophelines and their association with resistance to pyrethroids—A review. Parasit. Vectors.

[7] Du W (2005). Independent mutations in the Rdl locus confer dieldrin resistance to *Anopheles gambiae* and *An. arabiensis*. Insect Mol. Biol..

[8] Grau-Bové X (2021). Resistance to pirimiphos-methyl in West African *Anopheles* is spreading via duplication and introgression of the Ace1 locus. PLOS Genet..

[9] Assogba BS (2016). The ace-1 locus is amplified in all resistant *Anopheles gambiae* Mosquitoes: Fitness consequences of homogeneous and heterogeneous duplications. PLOS Biol..

[10] Yahouédo GA (2017). Contributions of cuticle permeability and enzyme detoxification to pyrethroid resistance in the major malaria vector *Anopheles gambiae*. Sci. Rep..

[11] Balabanidou V (2016). Cytochrome P450 associated with insecticide resistance catalyzes cuticular hydrocarbon production in *Anopheles gambiae*. Proc. Natl. Acad. Sci. U. S. A..

[12] Pelloquin B (2021). Overabundance of *Asaia* and *Serratia* bacteria is associated with Deltamethrin insecticide susceptibility in *Anopheles coluzzii* from Agboville, Côte d’Ivoire. Microbiol. Spectr..

[13] Omoke D (2021). Western Kenyan *Anopheles gambiae* showing intense permethrin resistance harbour distinct microbiota. Malar. J..

[14] Namias A, Jobe NB, Paaijmans KP, Huijben S (2021). The need for practical insecticide-resistance guidelines to effectively inform mosquito-borne disease control programs. Elife.

[15] Donnelly MJ, Isaacs AT, Weetman D (2016). Identification, validation and application of molecular diagnostics for insecticide resistance in malaria vectors. Trends Parasitol..

[16] Weill M (2004). The unique mutation in ace-1 giving high insecticide resistance is easily detectable in mosquito vectors. Insect Mol. Biol..

[17] Jones CM (2012). Footprints of positive selection associated with a mutation (N1575Y) in the voltage-gated sodium channel of *Anopheles gambiae*. Proc. Natl. Acad. Sci. U.S.A..

[18] Barrón MG (2019). A new species in the major malaria vector complex sheds light on reticulated species evolution. Sci. Rep..

[19] Erlank E, Koekemoer LL, Coetzee M (2018). The importance of morphological identification of African anopheline mosquitoes (Diptera: Culicidae) for malaria control programmes. Malar. J..

[20] Santolamazza F (2008). Insertion polymorphisms of SINE200 retrotransposons within speciation islands of *Anopheles gambiae* molecular forms. Malar. J..

[21] Moyes CL (2019). Analysis-ready datasets for insecticide resistance phenotype and genotype frequency in African malaria vectors. Sci. Data.

[22] Folmer O, Black M, Hoeh W, Lutz R, Vrijenhoek R (1994). DNA primers for amplification of mitochondrial cytochrome c oxidase subunit I from diverse metazoan invertebrates. Mol. Mar. Biol. Biotechnol..

[23] Besansky NJ (1994). Molecular phylogeny of the *Anopheles gambiae* complex suggests genetic introgression between principal malaria vectors. Proc. Natl. Acad. Sci..

[24] Folmer O, Black M, Hoeh W, Lutz R, Vrijenhoek R (1994). DNA primers for amplification of mitochondrial cytochrome c oxidase subunit I from diverse metazoan invertebrates. Aust. J. Zool..

[25] Scott JA, Brogdon WG, Collins FH (1993). Identification of single specimens of the *Anopheles gambiae* complex by the polymerase chain reaction. Am. J. Trop. Med. Hyg..

[26] Nag S (2017). High throughput resistance profiling of *Plasmodium falciparum* infections based on custom dual indexing and Illumina next generation sequencing-technology. Sci. Rep..

[27] Makunin A (2022). A targeted amplicon sequencing panel to simultaneously identify mosquito species and *Plasmodium* presence across the entire *Anopheles* genus. Mol. Ecol. Resour..

[28] Mavridis K (2018). Detection and monitoring of insecticide resistance mutations in *Anopheles gambiae*: Individual vs pooled specimens. Genes (Basel).

[29] Walsh IM, Bowman MA, Soto Santarriaga IF, Rodriguez A, Clark PL (2020). Synonymous codon substitutions perturb cotranslational protein folding in vivo and impair cell fitness. Proc. Natl. Acad. Sci. U.S.A..

[30] Miles A (2017). Genetic diversity of the African malaria vector *Anopheles gambiae*. Nature.

[31] Clarkson CS (2021). The genetic architecture of target-site resistance to pyrethroid insecticides in the African malaria vectors *Anopheles gambiae* and *Anopheles coluzzii*. Mol. Ecol..

[32] Jones CM (2012). Footprints of positive selection associated with a mutation (N1575Y) in the voltage-gated sodium channel of *Anopheles gambiae*. Proc. Natl. Acad. Sci. U. S. A..

[33] Riveron JM (2014). A single mutation in the GSTe2 gene allows tracking of metabolically based insecticide resistance in a major malaria vector. Genome Biol..

[34] Mitchell SN (2014). Metabolic and target-site mechanisms combine to confer strong DDT resistance in *Anopheles gambiae*. PLoS ONE.

[35] Menze BD (2016). Multiple insecticide resistance in the malaria vector *Anopheles funestus* from Northern Cameroon Is mediated by metabolic resistance alongside potential target site insensitivity mutations. PLoS ONE.

[36] Menze BD (2018). Bionomics and insecticides resistance profiling of malaria vectors at a selected site for experimental hut trials in central Cameroon. Malar. J..

[37] Asih PB (2012). Existence of the rdl mutant alleles among the anopheles malaria vector in Indonesia. Malar. J..

[38] Yang C, Huang Z, Li M, Feng X, Qiu X (2017). RDL mutations predict multiple insecticide resistance in *Anopheles sinensis* in Guangxi, China. Malar. J..

[39] Wondji CS (2011). Identification and distribution of a GABA receptor mutation conferring dieldrin resistance in the malaria vector *Anopheles funestus* in Africa. Insect Biochem. Mol. Biol..

[40] Grau-Bové X (2020). Evolution of the Insecticide Target Rdl in African *Anopheles* is driven by interspecific and interkaryotypic introgression. Mol. Biol. Evol..

[41] Casida JE, Durkin KA (2015). Novel GABA receptor pesticide targets. Pestic. Biochem. Physiol..

[42] Collins E (2019). The relationship between insecticide resistance, mosquito age and malaria prevalence in *Anopheles gambiae* s.l. from Guinea. Sci. Rep..

[43] Gen M (2021). An open dataset of *Plasmodium falciparum* genome variation in 7,000 worldwide samples. Wellcome Open Res..

[44] Oumbouke WA (2020). Fine scale spatial investigation of multiple insecticide resistance and underlying target-site and metabolic mechanisms in *Anopheles gambiae* in central Côte d’Ivoire. Sci. Rep..

[45] da Cruz DL (2021). First report of the L1014F kdr mutation in wild populations of *Anopheles arabiensis* in Cabo Verde, West Africa. Parasit. Vectors.

[46] Esling P, Lejzerowicz F, Pawlowski J (2015). Accurate multiplexing and filtering for high-throughput amplicon-sequencing. Nucleic Acids Res..

[47] Meiwald A (2020). Reduced long-lasting insecticidal net efficacy and pyrethroid insecticide resistance are associated with over-expression of CYP6P4, CYP6P3 and CYP6Z1 in populations of *Anopheles coluzzii* from South-East Côte d’Ivoire. J. Infect. Dis..

[48] Stica C (2019). Characterizing the molecular and metabolic mechanisms of insecticide resistance in *Anopheles gambiae* in Faranah, Guinea. Malar. J..

[49] da Cruz DL (2019). Detection of alleles associated with resistance to chemical insecticide in the malaria vector *Anopheles arabiensis* in Santiago, Cabo Verde. Malar. J..

[50] Orsborne J (2019). Investigating the blood-host plasticity and dispersal of *Anopheles coluzzii* using a novel field-based methodology. Parasit. Vectors.

[51] Coetzee M (2020). Key to the females of Afrotropical *Anopheles* mosquitoes (Diptera: Culicidae). Malar. J..

[52] Larsson A (2014). AliView: A fast and lightweight alignment viewer and editor for large datasets. Bioinformatics.

[53] Untergasser A (2012). Primer3—New capabilities and interfaces. Nucleic Acids Res..

[54] Boissière A (2013). Application of a qPCR assay in the investigation of susceptibility to malaria infection of the M and S molecular forms of *An. gambiae* s.s. in Cameroon. PLoS ONE.

[55] Li H, Durbin R (2010). Fast and accurate long-read alignment with Burrows–Wheeler transform. Bioinformatics.

