# Supplementary material for: High-throughput barcoding method for the genetic surveillance of insecticide resistance and species identification in Anopheles gambiae complex malaria vectors
Source: Sci Rep. 2022 Aug 16;12:13893. doi: 10.1038/s41598-022-17822-8 (PMC9381500; doi:10.1038/s41598-022-17822-8)
Supplement: Supplementary file 1 — Supplementary Information 1. [file 41598_2022_17822_MOESM1_ESM.docx]

**Supplementary Figures and Tables**

**Supplementary Figure 1.** Circular phylogenetic tree of the nuclear region *ITS2*. The tree was constructed using a maximum likelihood model (GTR + G) with a bootstrap node support of 100 replicates. Sample name is shown in the outer circle. The country of origin is indicated by color: pink (Guinea), green (Cabo Verde), brown (Ivory Coast) and blue (Kenya).


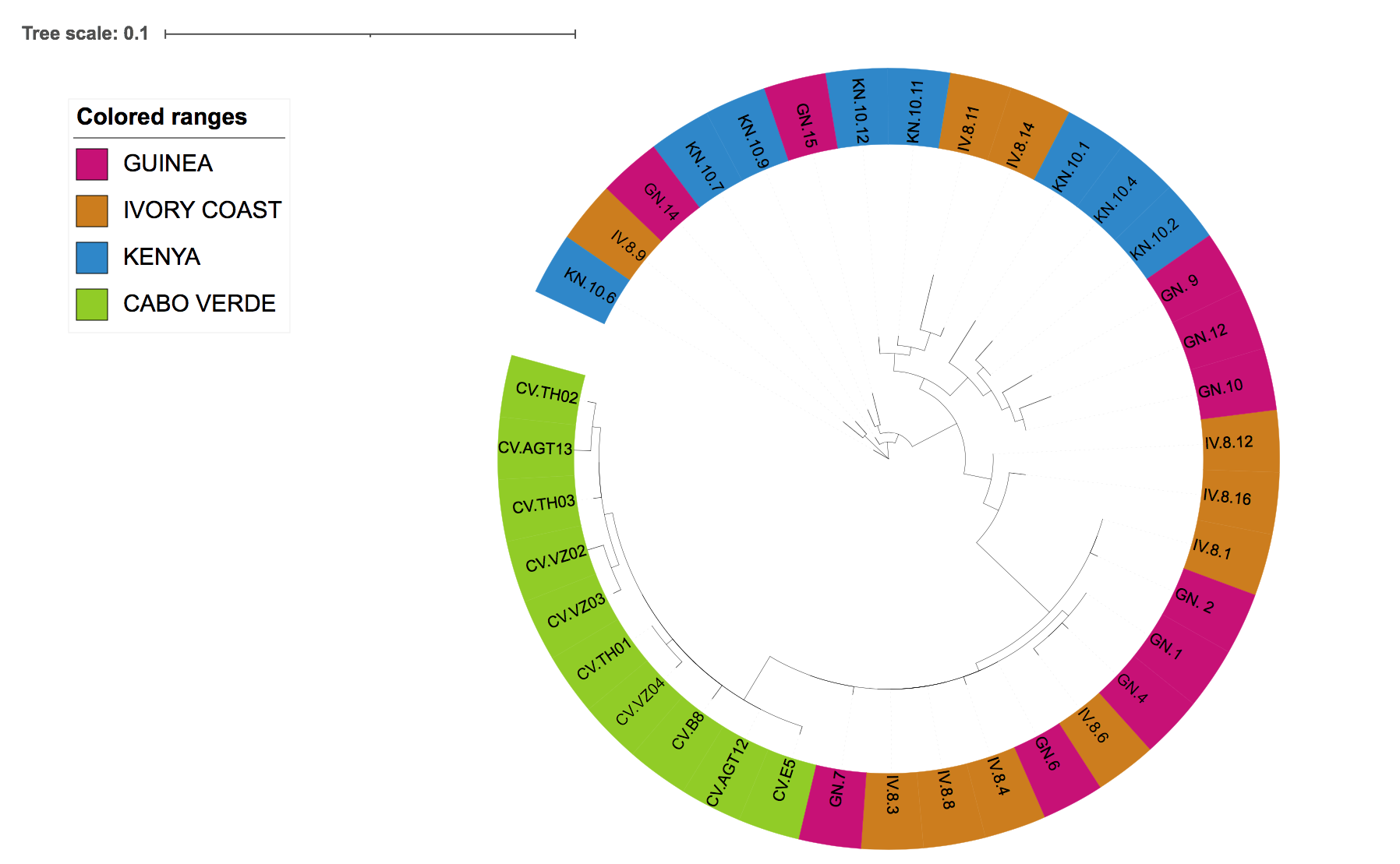


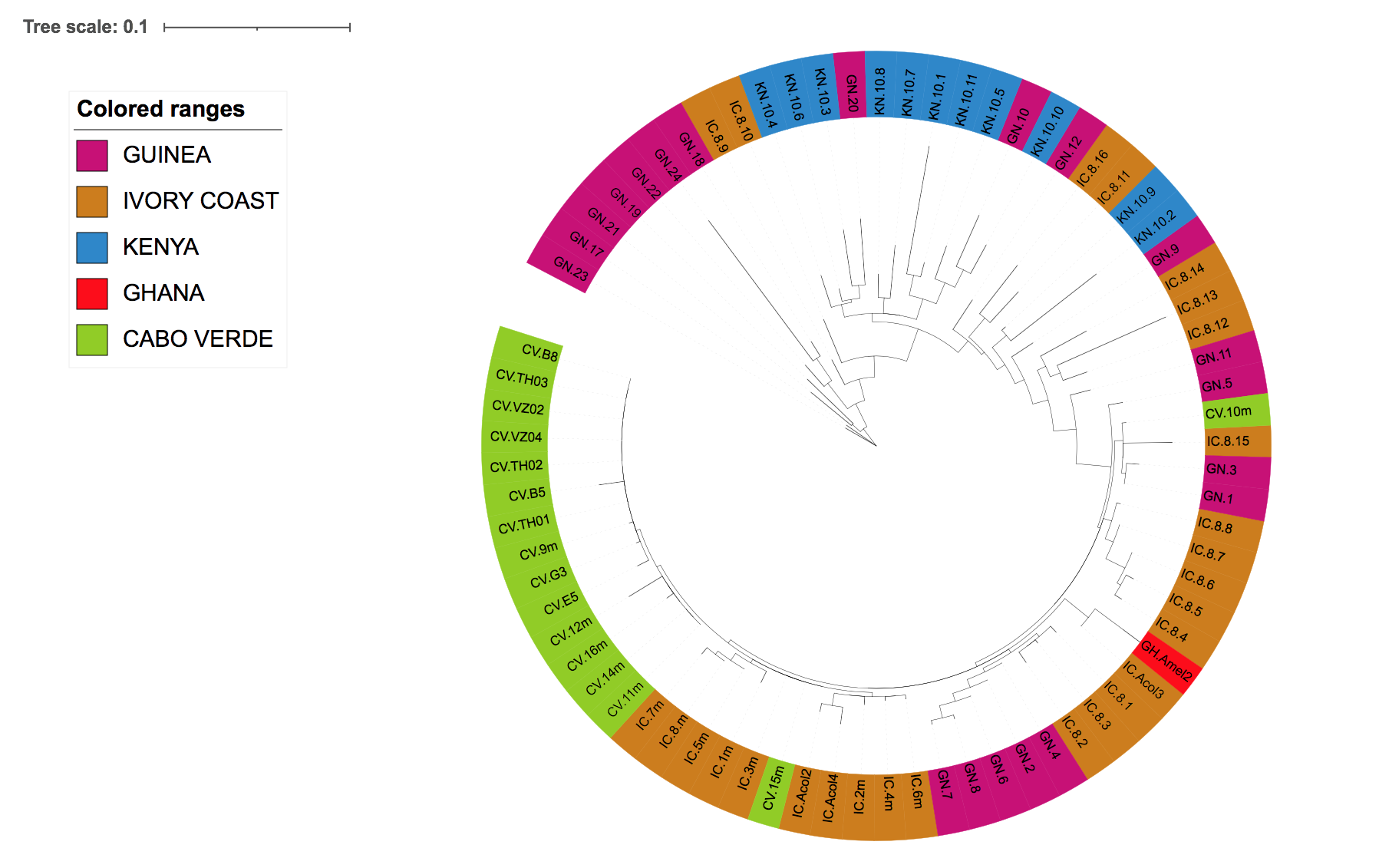


**Supplementary Figure 2**. Phylogenetic tree of the mitochondrial gene *mt-cox1*. The tree was constructed using a maximum likelihood model (GTR + G) with a bootstrap node support of 100 replicates. Sample name is shown in the outer circle. The country of origin is indicated by color: brown (Ivory Coast), green (Cabo Verde), red (Ghana), pink (Guinea) and blue (Kenya).


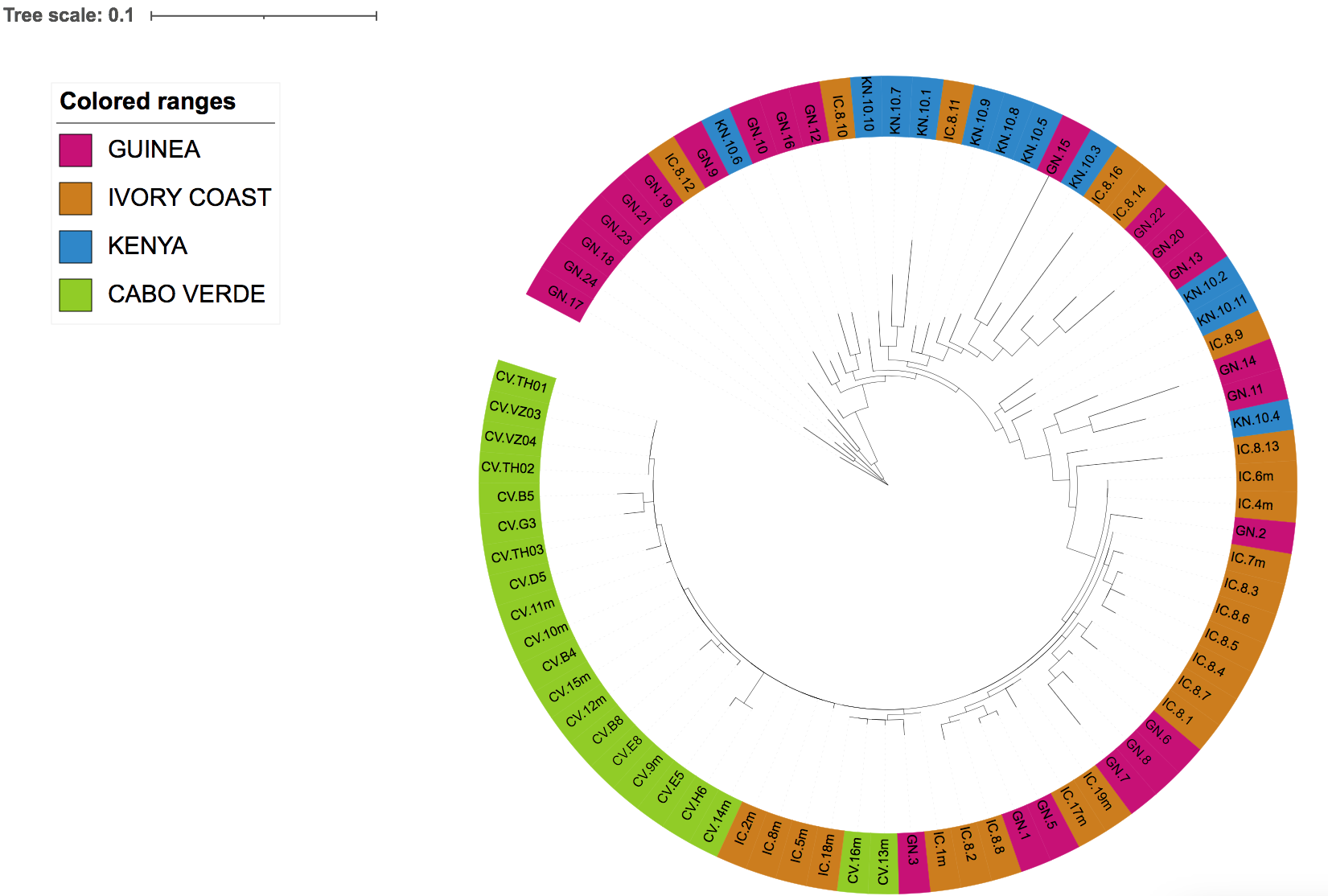


**Supplementary Figure 3**. Phylogenetic tree of the mitochondrial gene *mt-nd4*. The tree was constructed using a maximum likelihood model (GTR + G) with a bootstrap node support of 100 replicates. Sample name is shown in the outer circle. The country of origin is indicated by color: pink (Guinea), green (Cabo Verde), brown (Ivory Coast) and blue (Kenya).

**ITS2**


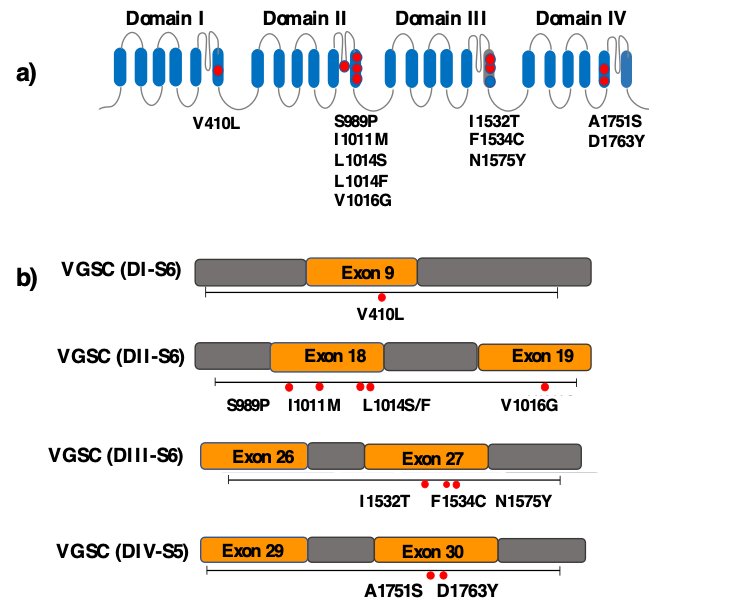


**Supplementary Figure 4**. Primer design for the voltage-sensitive sodium channel (vgsc) target gene. **(a)** The schematic diagram shows the sodium channel protein indicating the four internally homologous domains (I – IV), each having six hydrophobic transmembrane helices (blue). A total of 11 SNPs previously associated with knockdown resistance (kdr) were targeted (red dots). The respective amino acid substitutions are numbered according to the positions in Musca domestica. **(b)** For each protein domain (I-V), one set of primers was designed and named as vgsc (I-IV). The black bars show the selected regions for primer design and include introns (in grey) and exons (in orange). Exon numbering is in accordance with the vsgc in *Anopheles gambiae* Pest strain (AGAP004707-RA).


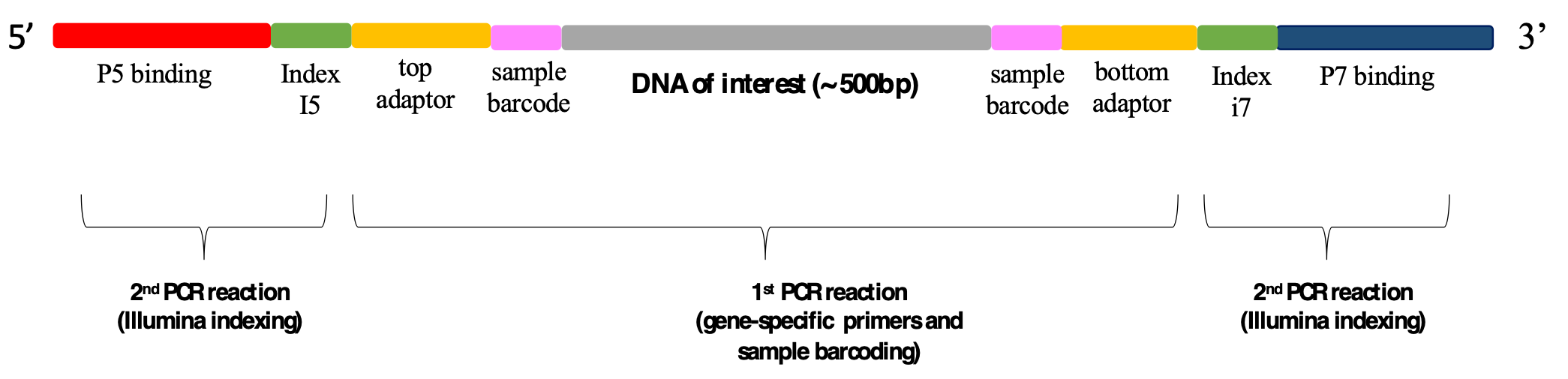


**Supplementary Figure 5.** Schematic of amplicon library. Schematic figure of amplicon library design highlighting the target genomic region (grey), In-line barcodes (6 nts) for sample multiplexing (pink), overhangs annealing site for the indexing primers (yellow), Illumina indexes (green) and P5 and P7 binding regions complementary to the oligos on the flow cell surface.


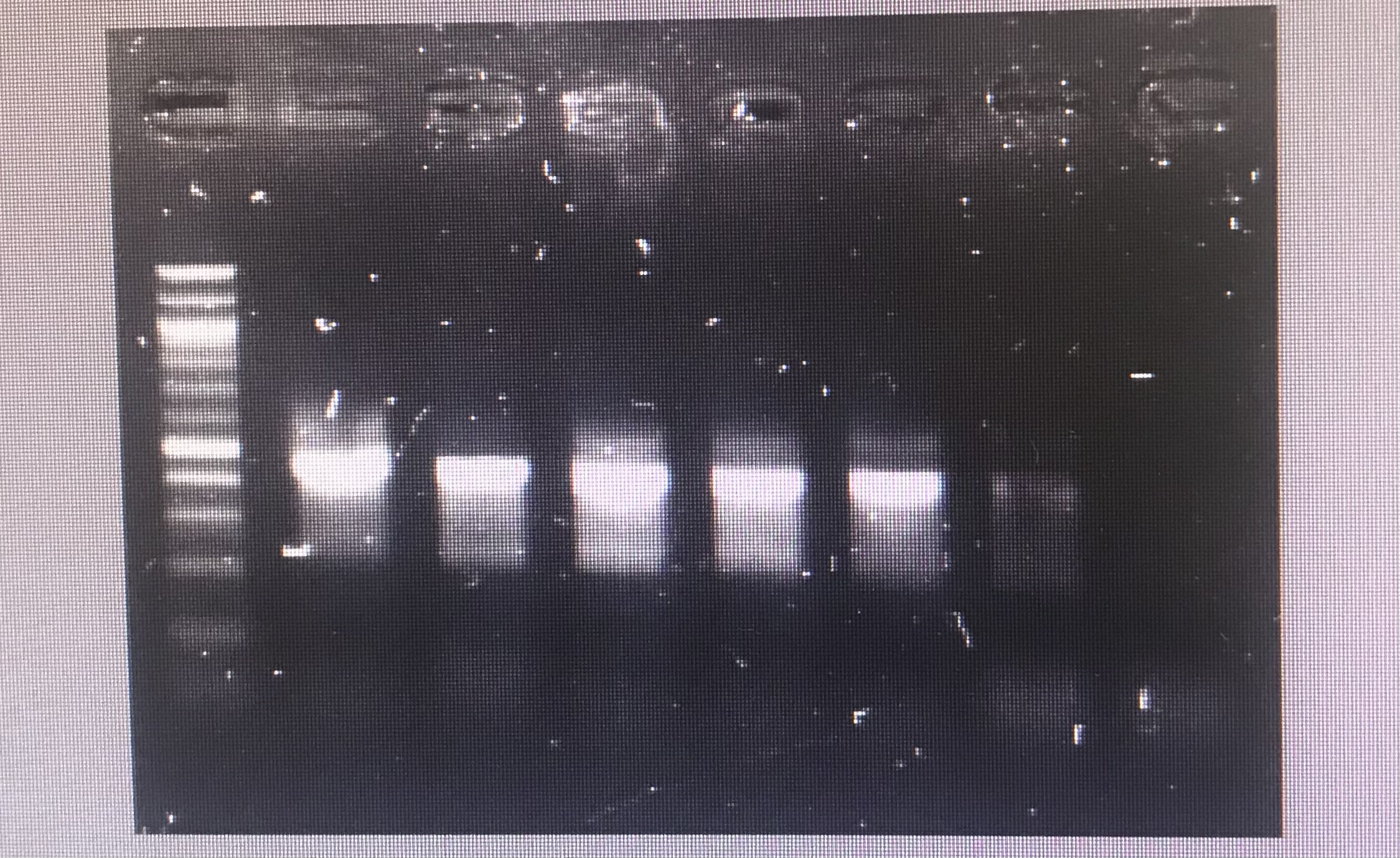


**Supplementary Figure 6**. **Amplification results of Plasmodium species**. In order: 100bp DNA Ladder; *P. falciparum sample 1* ;  *P. falciparum* (3D7 laboratory strain);  *P. malariae* *, P. ovale*, *P. vivax* sample 1;  *P. vivax* sample 2; negative control.

**Supplementary Tables**

| **Target gene*** |  | **Target sequences** | **Species calling** |
| --- | --- | --- | --- |
| SINE200 |  | TAGACGACTGTACTACGAGACCGGCCCATTT | *An. coluzzii* |
| SINE200 |  | CAACCGATGTCGGTCAAGGCCTGTCTGTAAC | *An. coluzzii* |
| SINE200 |  | GGCGGCGCGGTCTATTTGGGGATTGAACCCA | *An. coluzzii* |
| IGS |  | AGTGGATACTCTTGTGAGAGCAAGAGTGTAC | *An. melas* |
| IGS |  | GCCCCTTCCTCGATGGCGTTATTCACCATCT | *An. melas* |
| IGS |  | TTACACGAGTGGACAAGAGTTGGCTCCTTCC | *An. melas* |
| IGS |  | AGGGGATACTTTTGTGAGAGCAAGAGTGTAC | *An. gambiae s.s./ An. coluzzii* |
| IGS |  | GCCCCTTCCTCGATGGCGCAACGAACCATCT | *An. gambiae s.s./ An. coluzzii* |
| IGS |  | TTACACTAGTGGACAAGAGCTGGCTACTTCC | *An. gambiae s.s./ An. coluzzii* |
| IGS |  | AGGGGGATACTCTTGTGAGAGCATGAGTGTA | *An. arabiensis* |
| IGS |  | GCCCCTTCCTCGTTGGCGTAACTAACCATCT | *An. arabiensis* |
| IGS |  | TTATACTAGTGGACAAGAGTTGGCTACTTCC | *An. arabiensis* |
| GSTE2 |  | GACGTGACCTGTTTTCCCTTAAAAAGACTGAG | *An. arabiensis* |
| GSTE2 |  | GGACCGAGCATGACGATTGCCGACTTTAGCT | *An. arabiensis* |
| GSTE2 |  | CGGTGGCGGAGGCACCGATCTGGGCAAGTTT | *An. arabiensis* |

**Supplementary Table 1**. Selected nucleotide sequences for species identification. Sequences for species identification (*An. arabiensis*, *An. melas*, *An. gambiae s.s., An. coluzzii*).

* intergenic spacers (IGS), Short INterspersed Elements; ***An. coluzzii*

**Supplementary Tables 2-6 in excel file.**

|  | | | | | | |
| --- | --- | --- | --- | --- | --- | --- |
| **Primers name** | | **Primers sequences (5’-3’)** | **Target genes/genomic regions** | **Chromosome***  **(*An. gambiae* Pest)** | **Positions***  **(*An. gambiae* Pest)** | **Amplicon size (bp)** |
| IGS-universal fwd  IGS-GA reverse  IGS-ARA reverse  IGS- ME-reverse | | UN: GTGTGCCCCTTCCTCGATGT  GA: CTGGTTTGGTCGGCACGTTT  ARA: AAGTGTCCTTCTCCATCCTA  ME: TGACCAACCCACTCCCTTGA | 28S ribosomal RNA and rRNA intergenic spacer region | AgamP4_UNKN | 36384024-36384413 | *An. gambiae* (M,S) : 390  *An. melas*: 464  *An. arabiensis:* 315 |
| Sine200-fwd  Sine200-Rev | TCGCCTTAGACCTTGCGTTA  CGCTTCAAGAATTCGAGATAC | | SINE200 insertion  (Short INterspersed Elements) | AgamP4_X | 22951331-22951809 | *An. gambiae S* : 249  *An. gambiae M* : 479  *An. melas:* 249  *An. arabiensis:* 223 |
| ITS1-18S-Forward  ITS1-5.8S-Reverse | | TATGTTGGAGGTCAAGTGCG  TATCAATGTTCATGTGTCCTGC | Nuclear ribosomal Internal Transcribed Spacer 1 | AgamP4_UNKN | 31005267-31004850 | 420 |
| ITS2-5.8s-Forward  ITS2-28.S-Reverse | | GGCGCATCGGACGTTTA  CACTTGAGGCCTACGTG | Nuclear ribosomal Internal Transcribed Spacer 2 | AgamP4_UNKN | 35963328-35962837 | 492 |
| COXI-Forward  COX1-Reverse | | ATTACATTAGATCGAATACCATT  TAGTACAACTCCTGTTAGAC | Cytochrome c oxidase I | AgamP4_Mt | 1943-2491 | 549 |
| mt-ND4-Forward  mt-ND4-Reverse | | TCATAGTAATTCAAGACCAAC  GAAGCCCCAGTTTCTGG | Mitochondrial NADH dehydrogenase 4 | AgamP4_Mt | 8308-8824 | 517 |

**Supplementary Table 7** Primers for species identification and/or phylogenetic studies of *Anopheles gambiae* complex. The table shows the primer names and sequences (5’-3’), the chromosome and positions for the targeted genes or genomic regions and the amplicon size in base pairs (bp). Chromosome and positions are numbered according to the reference genome *Anopheles gambiae* Pest (GenBank: [CM000356](https://www.ncbi.nlm.nih.gov/nuccore/CM000356)-[CM000360](https://www.ncbi.nlm.nih.gov/nuccore/CM000360)).

| **gene** | **chromosome** | **pos genome** | **SNP** | **Amino acid change**  ***An. gambiae* (Pest)** | **Amino acid change**  ***Musca domestica*** |
| --- | --- | --- | --- | --- | --- |
| VGSC (DI-S6) | AgamP4_2L | 2391228 | G>C ,T | V402L | V410L |
| VGSC (DII-S6) | AgamP4_2L | 2422575 | T >C | S970P | S989P |
| VGSC (DII-S6) | AgamP4_2L | 2422643 | A>G | I992M | I1011M |
| VGSC (DII-S6) | AgamP4_2L | 2422651 | T >C | L995S | L1014S |
| VGSC (DII-S6) | AgamP4_2L | 2422652 | A>T | L995F | L1014F |
| VGSC (DII-S6) | AgamP4_2L | 2422657 | T>G | V997G | V1016G |
| VGSC (DIII-S6) | AgamP4_2L | 2429617 | T>C | I1527T | I1532T |
| VGSC (DIII-S6) | AgamP4_2L | 2429623 | T>G | F1529C | F1534C |
| VGSC (DIII-S6) | AgamP4_2L | 2429745 | A>T | N1570Y | N1575Y |
| VGSC (DIV-S5) | AgamP4_2L | 2430424 | G>T | A1746S | A1751S |
| VGSC (DIV-S5) | AgamP4_2L | 2430460 | G>T | D1758Y | D1763Y |
| Gste2 | AgamP4_3R | 28598166 | T>C | I114T | L114 |
| Gste2 | AgamP4_3R | 28598057 | C>G,A | F120 L | Y120 |
| Gste2 | AgamP4_3R | 28598062 | C>G | L119V | L119 |
| RDL | AgamP4_2L | 25429236 | C>G | A296G | A282G |
| RDL | AgamP4_2L | 25429235 | G>T | A296S | A282S |
| ACE1 | AgamP4_2R | 3492074 | G>A | G280S* | (G119S*) |

**Supplementary Table 8.** Targeted single nucleotide polymorphisms (SNPs) associated with insecticide resistance in the family *Culicidae* (Genus *Anopheles*, *Aedes* and *Culex*). Chromosomes, position of the SNPs and amino acid changes are numbered according to the reference genome *Anopheles gambiae* Pest. Amino acid changes are also numbered according to *Musca domestica*. * Amino acid changes are also numbered according to *Torpedo californica*.

| **Primers name** | **Primers sequences (5’-3’)** | **Target genes/genomic regions** | **Chromosome*** | **Positions*** | **Amplicon size (bp)** |
| --- | --- | --- | --- | --- | --- |
| VGSCI Fwd  VGSC-I Rev | ATTCGTTATTCTTCAGATGAACT  ATTCTCACCCGAAGTGC | Voltage gate sodium channel  Domain I-Subunit 6 | AgamP4_2L | 2390813-2391328 | 517 |
| VGSC-II Fwd  VGSC-II Rev | GTTTTGCTAGCCTAATTGC  TGTCGGTTGAACGGATGCTATT | Voltage gate sodium channel  Domain II-Subunit 6 | AgamP4_2L | 2422417-2422919 | 503 |
| VGSC-III Fwd  VGSC-III Rev | TTCATGGGAAAATTCACCAA  AATTAGTGCTCCAAACACAAAC | Voltage gate sodium channel  Domain III-Subunit 6 | AgamP4_2L | 2429356-2429845 | 490 |
| VGSC-IV Fwd  VGSC-IV Rev | CGAGCCATGGAATTTGT  TGATGTGATCCAGTTACAGA | Voltage gate sodium channel  Domain IV-Subunit 5 | AgamP4_2L | 2430093-2430593 | 501 |
| ACE1-Forward  ACE1-Reverse | CTGGTGGTCAACACGGA  GAACAGTCCCGCATTGC | Acetylcholinesterase 1 | AgamP4_2R | 3491732-3492229 | 498 |
| GSTe2-fwd  GSTe2-rev | GCCCGGATGAGATTCAT  TTCCAAATGCTTCCAAATTT | Glutathione S- transferase 2 | AgamP4_3R | 28598182-28597684 | 485 |
| RDl-Fwd  RDl-Rev | CATTGCAATCATCACCATCA  CCAGCAGACTGGCAAATACC | Resistance to dieldrin (RDL) gaba receptor | AgamP4_2L | 25428861-25429373 | 500 |
| *Plasmodium-*fwd  *Plasmodium*-rev | ACGATCAGATACCGTCGTAATCTT  CAGGTTAAGATCTCGTTCG | 18S ribosomal RNA | LR131493.1  chr13 | 2699402 - 2699797 | 396 |

**Supplementary Table 9.** Primers for insecticide resistance genes in *Anopheles gambiae* complex and diagnosis of *Plasmodium* sp infections. Primers sequences, chromosome and positions for each target gene or genomic regions are shown and includes: 4 domains of the voltage-gated sodium channel gene (*vgsc* 1-4), acetylcholinesterase 1(*ace1*), glutathione S- transferase 2 (*gste2*), resistance to dieldrin (*rdl*) gaba (gamma-amino butyric Acid) receptor. The 18s ribosomal RNA of *Plasmodium* ssp: *P. malariae* (M54897.1), *P. ovale* (MG641069.1) *P. falciparum* (XR_002273081.2) and *P. vivax* (XR_003001206.1) were aligned for the design of universal primers. ^*^ Chromosomes and SNPs positions are numbered according to the reference genomes *Anopheles gambiae* Pest and *Plasmodium falciparum* PF3D7.

|  | **Illumina Tail** | **Barcode** |
| --- | --- | --- |
| B1 | ACACTCTTTCCCTACACGACGCTCTTCCGATCT | ATCACG |
| B2 | ACACTCTTTCCCTACACGACGCTCTTCCGATCT | CGATGT |
| B3 | ACACTCTTTCCCTACACGACGCTCTTCCGATCT | TTAGGC |
| B4 | ACACTCTTTCCCTACACGACGCTCTTCCGATCT | TGACCA |
| B5 | ACACTCTTTCCCTACACGACGCTCTTCCGATCT | ACATCT |
| B6 | ACACTCTTTCCCTACACGACGCTCTTCCGATCT | TGCCAA |
| B7 | ACACTCTTTCCCTACACGACGCTCTTCCGATCT | AGCTCG |
| B8 | ACACTCTTTCCCTACACGACGCTCTTCCGATCT | ACGTCA |
| B9 | ACACTCTTTCCCTACACGACGCTCTTCCGATCT | GCAGAT |
| B10 | ACACTCTTTCCCTACACGACGCTCTTCCGATCT | GATCAC |
| **Added to reverse primer (5’-3’)** | **Illumina Tail** | **Barcode** |
| BR1 | GACTGGAGTTCAGACGTGTGCTCTTCCGATCT | CAGATC |
| BR2 | GACTGGAGTTCAGACGTGTGCTCTTCCGATCT | ACTTGA |
| BR3 | GACTGGAGTTCAGACGTGTGCTCTTCCGATCT | GTTCAG |
| BR4 | GACTGGAGTTCAGACGTGTGCTCTTCCGATCT | TAGCTT |
| BR5 | GACTGGAGTTCAGACGTGTGCTCTTCCGATCT | GGCTAG |
| BR6 | GACTGGAGTTCAGACGTGTGCTCTTCCGATCT | CTTGTA |
| BR7 | GACTGGAGTTCAGACGTGTGCTCTTCCGATCT | TGAGAT |
| BR8 | GACTGGAGTTCAGACGTGTGCTCTTCCGATCT | ATGTGC |
| BR9 | GACTGGAGTTCAGACGTGTGCTCTTCCGATCT | GTATCA |
| BR10 | GACTGGAGTTCAGACGTGTGCTCTTCCGATCT | CTACTG |

**Supplementary Table 10**. DNA in-line barcodes and Illumina tails added to the 5’ end of the forward and reverse primers before PCR amplification.
